# Diagnosis and treatment of chronic cough in China: an insight into the status quo

**DOI:** 10.1186/1745-9974-8-4

**Published:** 2012-07-28

**Authors:** Kefang Lai, Wei Luo, Guangqiao Zeng, Nanshan Zhong

**Affiliations:** 1 State Key Laboratory of Respiratory Diseases, 1st Affiliated Hospital, Guangzhou Medical College, Guangzhou, GZ, China; 2 State Key Laboratory of Respiratory Diseases, 1st Affiliated Hospital, Guangzhou Medical College, Guangzhou, GZ, China; 3 State Key Laboratory of Respiratory Diseases, 1st Affiliated Hospital, Guangzhou Medical College, Guangzhou, GZ, China; 4 State Key Laboratory of Respiratory Diseases, 1st Affiliated Hospital, Guangzhou Medical College, Guangzhou, GZ, China

**Keywords:** Airway inflammation, Chronic cough, Diagnosis, Epidemiology, Pathogenesis

## Abstract

Chronic cough is a very common complaint in clinics throughout China. Clinical and basic science research on chronic cough started late, but in recent years the effort has yielded promising findings regarding the etiological diagnosis, treatment and pathogenesis. We found that inflammation in nonasthmatic eosinophilic bronchitis has some similarities to cough variant asthma but also a number of distinct differences. Recent evidence has also suggested a mechanistic link between airway neurogenic inflammation and and gastroesophageal reflux cough (GERC). Cough-related animal models have been developed, including models for esophageal reflux, nonasthmatic eosinophilic bronchitis and allergic rhinitis. Normal reference values for differential cell counts in induced sputum, cough sensitivity and esophageal 24-h pH monitoring in Chinese healthy subjects have been established. By using a modified algorithm for the etiological diagnosis of chronic cough, the causes of chronic cough have been investigated across a number of cities in China. The most common causes of chronic cough are cough variant asthma, eosinophilic bronchitis, upper airway cough symptoms, atopic cough and GERC, however, there are some regional variations. The Chinese National Guidelines on Diagnosis and Management of Chronic Cough were drafted in 2005, updated in 2009, and have been widely publicized and disseminated through many channels since their publication.

## Review

In the clinical setting, chronic cough is defined as cough being sole or predominant symptom and lasting for more than 8 weeks with normal chest X-ray. Chronic cough is a common complaint in China, as it is in Europe, America and Japan. An epidemiological study demonstrated a 3.3% incidence of chronic cough among college students in Guangzhou [1]. It is also estimated that patients who seek medical attention for chronic cough account for 30-40% of the visits to respiratory clinics in China. However, this condition has frequently been misdiagnosed and inappropriately treated. In a clinical survey of chronic cough patients, Lai et al. showed that 81% of patients with chronic cough had been misdiagnosed with chronic bronchitis, pharyngitis, or laryngitis, and 93% had been treated with antibiotics and/or antitussives [2]. Misdiagnosis and inappropriate treatment of chronic cough substantially interferes with quality of life in the majority of patients. For example, nearly 50% of female patients were found to have urinary incontinence [3].

Since the description of an anatomy-based diagnostic protocol for chronic cough in 1977 by Irwin and colleagues, many studies have been undertaken in America, Europe and Japan, concerning the pathogenesis, etiologic diagnosis and management of chronic cough with the subsequent development of national guidelines [4-6]. Since 2005, similar studies on local populations in China have yielded promising findings. Here we describe these research efforts in particular the findings on the pathogenesis, etiology, diagnosis and treatment of cough in China together with cough guideline development and dissemination.

## Airway inflammation in chronic cough due to different etiology

### Airway inflammation in eosinophilic bronchitis (EB) and cough variant asthma (CVA)

Airway inflammation in EB shares some similarities to asthma regarding the recruitment of inflammatory cells such as eosinophils (Eos), T lymphocytes and mast cells, as detected using the induced sputum test, bronchoalveolar lavage fluid (BALF) cytology and airway mucosal biopsy. In addition, airway inflammation is attributable to the pro-inflammatory mediators released from these cells, which include leukotriene C4 (LTC_4_), histamine, prostaglandins, and eosinophilic cationic protein (ECP). Despite the similarities between EB and asthma, there are a number of disparities:

#### Airway mucosal inflammation

As shown in one of our previous studies, EB, CVA and classic asthma are airway inflammatory diseases characterized by eosinophilic infiltration, however, the percentage of Eos in induced sputum differs significantly in the three conditions, ranging from 0.113 ± 0.147% in EB to 0.190 ± 0.180% in CVA and to 0.386 ± 0.267% in classic asthma. In addition, Luo and colleagues reported that Eos was rarely found in the BALF from EB patients [7]. These findings indicate that the inflammation in EB may be largely restricted to central airways only, unlike that in CVA and classic asthma. Luo et al. identified infiltrating mast cells and Eos in the airway submucosa of EB patients, and the infiltration intensity of Eos was significantly lower than that found in CVA patients [8], indicating that reduced inflammatory infiltration may be linked to the absence of airway hyperresponsiveness in EB.

#### Airway remodeling

The thickness of the airway basement membrane is increased in both EB and CVA as compared with healthy controls, however, it remains to be determined whether there is a difference between CVA and classic asthma [8,9]. Li et al. demonstrated thickening of the basement membrane and the presence of mucosal and submucosal cells which stained positive for transforming growth factor β1 (TGFβ1) or platelet-derived growth factor (PDGF) in EB, although with a milder severity or fewer cells compared with the findings in asthma. These results suggest that TGFβ1 and PDGF may also play an important role in airway submucosal fibrosis in EB patients [10].

#### Inflammatory mediators and cytokines

Higher levels of LTC4 in induced sputum, histamine and 8-iso-prostaglandin in BALF were found in CVA patients compared with EB patients [7,11]. Similar to the results presented by Sastre et al. [12], our studies also showed higher levels of prostaglandin E2 (PGE2) in induced sputum from EB patients than in CVA or asthmatic patients. Taking into consideration the elevated LTC4 levels in asthmatic patients [7,11,13], it is speculated that the over-expression of PGE2 and lower LTC_4_/PGE2 ratio in induced sputum may represent the inflammatory basis for the absence of airway hyperresponsiveness in EB patients.

Th2-driven airway inflammation was identified in patients with comparable increased levels of corresponding cytokines (such as IL-4, IL-5, and IL-13) in induced sputum in both EB and asthma [12,13]. However, other studies have demonstrated significant differences in IL-13 level in induced sputum and airway mucosa, as well as in the levels of certain chemokines (such as CXCL8 and CXCL10) in BALF between EB and asthma [14,15]. The reasons for these mixed findings are unclear. In addition, evidence has shown that the levels of nerve growth factor (NGF) and IL-4 in induced sputum in CVA patients were significantly higher than those in healthy controls [16], suggesting that a nerve-immune mechanism is closely related to the eosinophilic inflammation in CVA, where NGF and IL-4 may be involved as mediators.

### Airway inflammation in gastroesophageal reflux-related cough (GERC)

#### Esophageal-tracheal reflex and neurogenic inflammation

Liu et al. found that gastroesophageal reflux is an independent cause of cough, and that reflux of the distal esophagus may induce cough in most cases with GERC. In patients with GERC, distal reflux episodes accounted for 88.23% of all reflux events during 24-hour pH monitoring, and were positively correlated to the onset of coughs [17]. In guinea pig models, esophageal hydrochloric acid (HCl) instillation resulted in increased plasma extravasation in the airway, which was apparently inhibited with neural endopeptidase or bilateral vagotomy. Therefore, it was speculated that esophageal-tracheal reflux may be a mechanism underlying the induction of cough [18]. In other studies, repeated instillation of HCL into the distal esophagus increased resistance but did not alter airway responsiveness in guinea pigs, and was accompanied by up-regulation of NGF and substance P (SP) in the airway mucosa [19-21]. Higher levels of SP and calcitonin gene-related peptide (CGRP) in the supernatant of induced sputum, increased numbers of SP positive-induced-sputum cells, and enhanced expression of SP were observed in the airway mucosa, as compared with normal controls and patients with gastroesophageal reflux but without cough (GERD) [22]. In these animal and clinical studies, neurogenic inflammation was closely correlated with the occurrence of GERC. In addition to airway neuropeptides, activation of mast cells in the lower respiratory tract was also indicated to be a possible mechanism underlying GERC [23].

## Establishment of a cough-related animal model

Animal cough models include models of isolated cough and those of cough-related diseases. Several specific cough-related animal models currently available in China are described below.

### Animal models of esophageal reflux

Animal models of reflux esophagitis with concomitant inflammation in the tracheal and bronchial mucosa were successfully established in guinea pigs by repeated esophageal infusion of HCL and pepsin. These animal models provide a basis for studies on the pathogenesis of reflux-related respiratory disorders [24].

### Animal models of eosinophilic bronchitis

An eosinophilic bronchitis model in BALB/c mice has been established for the first time. To establish the model of this kind, all the mice were sensitized by intraperitoneal ovalbumin (OVA) and subsequently challenged by either intranasal drip or inhalation of OVA [25]. This model is characterized by eosinophilic inflammation, absence of airway hyperresponsiveness, increased cough reflex sensitivity and response to corticosteroids. It represents a valuable tool for in-depth investigations into the pathogenesis and airway responsiveness in EB.

### Animal models of allergic rhinitis (AR)

An allergic rhinitis model was successfully established by sensitizing mice with intraperitoneal OVA and then challenging them with an intranasal OVA drip [26]. In addition, a simple method to measure nasal airway resistance and response was developed. Using this method, we found that (1) nasal resistance accounted for more than 50% of the total airway resistance; (2) nasal resistance and response were markedly increased in allergic rhinitis mice; and (3) mucosal swelling may contribute to the higher nasal airway resistance in mouse models of AR [26].

## Etiologies of chronic cough in China

### Common causes

Early studies in Europe and America indicated that the most common causes of chronic cough in specialist clinics were postnasal drip syndrome (PNDS), bronchial asthma and gastroesophageal reflux (GER), which accounted for more than 93.6% of all causes [27]. Referring to the anatomic-based diagnostic protocol developed by Irwin and colleagues, we introduced the induced sputum test in the diagnostic protocol which has been in widespread use in China. Ma et al. investigated the causes of chronic cough in 86 patients in Guangzhou, and showed that EB accounted for 15.1% of etiologies [28]. Lai et al. performed a subsequent investigation on the etiological diagnosis of chronic cough, and the results revealed that EB was the most common cause of chronic cough (22%), followed by PNDS (17%), CVA (14%), GER (12%) and atopic cough (AC, 12%) [29]. However, studies in Beijing [30] and Shanghai [31] showed a dramatically lower frequency of EB as a cause of chronic cough (Table 1). It remains to be determined whether such a difference arises from disparity in geographic factors or in diagnostic approaches. 

**Table 1:** Causes of chronic cough in different regions of China

	**Regions**	**N**	**Asthma**	**PNDS**	**GERC**	**EB**	**Others**
Ma et al. [28] (2003)	Guangzhou	86	28%	26%	14%	15%	/
Lai et al. [29] (2006)	Guangzhou	194	14%	17%	12%	22%	AC (12%)
Wang et al. [30] (2007)	Beijing	106	66.3%	14.1%	10.4%	1.9%	PIC (3.8%)
Yang et al. [31] (2005)	Shanghai	105	51.4%	26.7%	1.9%	5.7%	PIC (8.5%)
Cao et al. [32] (2009)	Chongqing	233	25.5%	44.4%	9.1%	/	PIC (2.1%)
Si et al. [33] (2010)	Shenyang	96	39.4%	13.5%	1.9%	12.5%	AC (11.5%)

### Atopic cough (AC)

The definition of atopic cough was first proposed in 1989 by Fujimura [34]. In Japan, AC is defined as a bronchodilator resistant non-productive cough with sputum eosinophilia or an atopic constitution (e.g. positive allergen skin tests, high levels of total IgE or positive specific IgE and increased percentage of blood eosinophils) [35]. In contrast to the Japanese definition, sputum eosinophilia is not included in the Chinese criteria for AC. As a result, no overlap exists in the etiological diagnosis between EB and AC in China [36,37], although increased cough sensitivity, a component of AC using the Japanese definition, does not occur exclusively in AC, and may be absent in a percentage of Chinese patients with AC. Accordingly, it could not be regarded as a requirement for the diagnosis of this condition in China. Clinically, we have also identified a number of patients who presented with features of AC and showed satisfactory response to steroids and anti-histamines, despite controversy over the diagnosis and pathogenesis. Our results showed that AC accounts for 12% of all patients with chronic cough [29].

### Uncommon causes of chronic cough presenting to specialist clinics

Our previous study showed that about 5% of chronic cough can be attributed to chronic bronchitis [29]. Chronic cough due to bronchial tuberculosis is not a rare condition in China, where pulmonary tuberculosis is highly prevalent. While the majority of these patients showed clinical signs or imaging findings of complicating pulmonary tuberculosis, certain cases of simple bronchial tuberculosis may be manifested predominantly or solely by chronic cough. In Chongqing, bronchial tuberculosis was shown to account for chronic cough in 6% of the patients observed by Bi and his colleagues [38]. In addition, bronchiectasis-induced chronic cough is not uncommon. In southern Fujian Province, a study on 342 patients with chronic cough revealed that bronchiectasis was causative of the condition in 1.4% of the participants [39]. In the elderly, ACEI-induced cough should be taken into consideration as a common cause of chronic cough, apart from CVA and PNDS [40]. In recent years, a few other rare causes of chronic cough were also identified, such as cervical spondylosis and heterotopic salivary gland at the base of the tongue [41,42].

## Diagnosis of chronic cough

### Common tests for chronic cough

#### Differential cell counts in induced sputum

In 2003, analysis of differential cell count in induced sputum samples was introduced into the diagnostic protocol for chronic cough in China [43]. This test does not require sophisticated instruments, and has been proved to be an essential tool in identifying EB, the common cause of chronic cough. In 2004, this test was officially used as a routine clinical investigation, and is recommended as a first-line test in the Chinese National Guidelines on Diagnosis and Management of Chronic Cough [37]. Sputum induction and differential cell analysis is now accessible in nearly 200 medical centers in China, and plays an important role in the etiological diagnosis of chronic cough. In 2007, the normal reference values of induced sputum cytology in Guangzhou were established in a pilot study of 117 healthy volunteers (Eos <2.5% and neutrophils <50%) [44].

#### Assessment of cough sensitivity

In China, the assessment of cough sensitivity is not yet an established part of the diagnostic algorithm for chronic cough, and is available in only a few medical institutions. The normal lower limit of cough sensitivity has been primarily established (C5 ≥62.5 μmol/L) using a single metered-dose inhalation of capsaicin [45]. Increased cough sensitivity has been detected in many patients with chronic cough albeit arising from a wide variety of etiologies [46]. Shi et al. recruited 104 patients with a confirmed single cause of chronic cough, and higher cough sensitivity to capsaicin was found in patients with chronic cough as compared with healthy controls, but there was no significant difference in cough sensitivity across the different causes of chronic cough [47]. However, Chen et al. found that cough sensitivity in patients with postinfectious cough, GERC and EB was markedly higher than that in patients with CVA and asthma as well as in healthy controls [45,48,49].

#### Detection of airway hyperresponsiveness

In the Chinese National Guidelines on Diagnosis and Management of Cough, detection using spirometry and airway hyperresponsiveness is recommended as the first-line tests for the diagnosis of chronic cough [50]. While spirometry is usually normal in patients referred with chronic cough, measurement of airway hyperresponsiveness is essential for the diagnosis of CVA. Lung function tests are largely available in tertiary hospitals (in particular, the grade-A tertiary hospitals) throughout China, where spirometry is fully accessible. In most of these institutions, the bronchodilator test (74.1%) and bronchial provocation test (65.1%) are also used [51].

#### 24-h esophageal pH monitoring

Ambulatory 24-h esophageal pH monitoring has some value in the diagnosis of gastroesophageal reflux disease and to assess the association between reflux events and cough episodes. The normal Demeester score is <12.70 for healthy Chinese subjects [52], slightly lower than reported elsewhere (14.72) [53]. In the Chinese National Guidelines on Diagnosis and Management of Cough [54], Demeester score of ≥12.70 and SAP of ≥75% are measures diagnostic of GERC. Owing to its high costs in relation to devices and supplies, 24-h esophageal pH monitoring is not currently in widespread use in China, even in some of the large hospitals.

### Diagnostic algorithm

**Figure 1:**
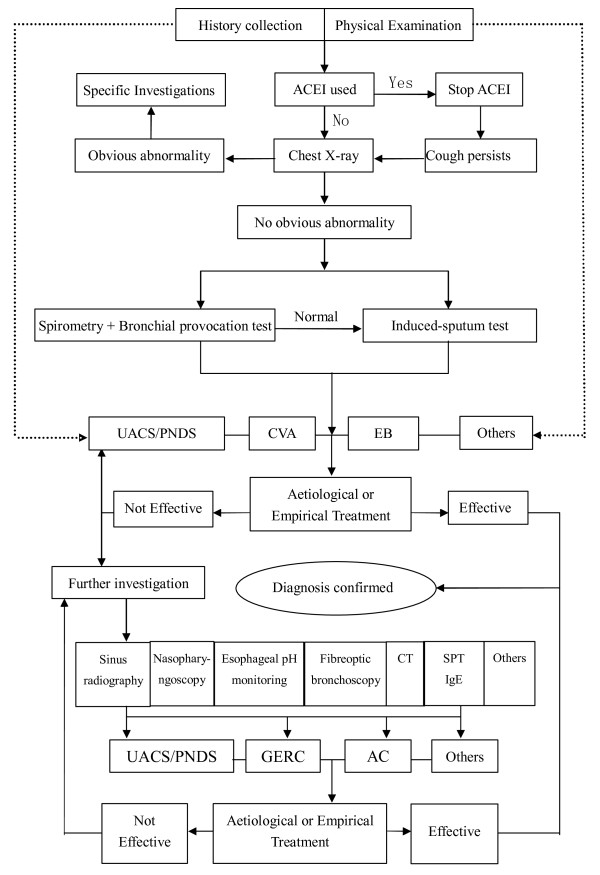
Algorithm for etiological diagnosis of chronic cough.

NB: 1) ACEI: angiotensin converting enzyme inhibitor; UACS: upper airway cough syndrome; PNDS: postnasal drip syndrome; CVA: cough variant asthma; EB: eosinophilic bronchitis; SPT: skin prick test; IgE: immunoglobulin E; GERC: Gastro-esophageal reflux-related cough; AC: atopic cough. 2) Empirical treatment can be initiated in grass-root institutions or to individuals with low income. Patients who fail empirical treatment should be referred for thorough investigations of underlying etiologies at institutions with sufficient access to medical resources, to prevent a delay in treatment.

The algorithm for the etiological diagnosis of chronic cough has been re-structured to adapt to the clinical picture of this condition in China (Figure 1), with reference to the diagnostic protocols in the ACCP and ERS guidelines [54]. The new algorithm was developed to incorporate the following principles: (1) Focus on medical history (including ENT and gastrointestinal diseases) and physical workup can give rise to a narrow spectrum of diagnostic clues; (2) Laboratory investigations are ordered on the basis of history, beginning with simple and progressing to more complex testing. As such, induced sputum, spirometry and bronchial provocation test are recommended as first-line tests. 24-h esophageal pH monitoring is considered only after the first-line tests fail to produce a diagnosis, or, when the patient does not respond to initial treatments or presents with reflux-related symptoms. Empirical therapy may be initiated if 24-h esophageal pH monitoring is not available. If these tests are non-confirmatory, or the patient does not respond to GERC-targeted empirical therapy, High-resolution computed tomography(HRCT), bronchoscopy or cardiac examination should be considered to rule out rare causes of cough, including extrapulmonary diseases. (3) Chronic cough-related tests can be performed at the same time or in sequence. A diagnostic trial of therapy can be given where tests for chronic cough are not fully available, so that the cause of cough can be identified according to treatment response. In patients who partially respond to treatment, physicians should be consider the possibility of more than one cause for the cough. 

## Development and dissemination of Chinese national guidelines on diagnosis and management of chronic cough

Along with increasing concern in this field, a series of studies addressing the etiologies and treatment of cough have been conducted in Europe, America and Japan over the past 30 years. These efforts have shed light on the common causes of chronic cough and have given rise to the development of guidelines on the diagnosis and treatment of cough [4-6]. In China, the study of chronic cough started late, but has yielded promising findings providing important insights into the etiological diagnosis, treatment and pathogenesis of chronic cough. In order to strengthen and standardize the etiological diagnosis and treatment of cough, and to promote clinical studies on cough, in particular chronic cough, the Asthma Workgroup of Respiratory Society, Chinese Medical Association in 2005 commissioned a panel of experts to draft the early edition of the Chinese National Guidelines on Diagnosis and Management of Cough, based on a detailed review of international and national evidence from clinical studies on this subject and following a philosophy of “integrated coverage, highlighted aspects and intended practicability”. These guidelines concentrate on the classification, etiologies, diagnosis and management of cough. To refine those guidelines, and to acknowledge the latest progress in cough studies within and outside China, an updated edition was published in 2009 [54], followed by an English version released in 2011 [50]. Experts from other disciplines, such as otorhinolaryngology and gastroenterology, were invited to participate in the revision of the guidelines to ensure the quality and practicability of the document. While the full-text of this revised edition basically preserves the structure of the 2005-released Guidelines, two sections — subacute cough (focusing on post-infectious cough) and the empirical treatment of chronic cough, have been added. In the section on empirical treatment, the therapeutic principles suggested that: (1) empirical therapy should target all common causes of chronic cough; (2) steroid-responsive disorders (CVA, EB and AC) are common causes of chronic cough in China, and (3) potential causes of chronic cough can be indicated based on specific medical history. For instance, in patients with acid reflux-related symptoms, the therapeutic method used for GERC should be taken into account; in patients with atopy or nocturnal cough, treatment aimed at CVA/EB or AC should be considered first. These practices may be more adaptable in community or rural hospitals.

Since their release, the Chinese cough guidelines have been widely publicized and disseminated through journals, newsletters, conferences, internet and many other channels. A Cough Forum has been held annually around China since 2007, which provides a platform to discuss the cough research hot topics. A cough symposium has also been added to the agenda of the Annual Conference of Chinese Society of Respiratory Diseases. In addition, several reputable national journals (such as the Chinese Journal of Tuberculosis and Respiratory Diseases, Chinese Journal of Respiratory and Critical Care Medicine, and International Respiratory Diseases) have launched columns or published special issues on chronic cough to introduce the state-of-the-art advances in this field worldwide. Furthermore, keynote speakers from America, Europe and Japan are invited to China on a regular basis to share their expertise on chronic cough.

Looking to the future, we still have a long way to go until the mystery surrounding chronic cough is unraveled. The closer communication and collaborations between Chinese and international specialists will improve our understanding of the diagnosis and treatment of chronic cough in the future.

## Conclusions

Chronic cough is a common complaint in the clinic in China. In the last decade, the etiological diagnosis and pathogenesis of chronic cough have been widely studied in China, and promising findings have been reported. The pathophysiology and causes of chronic cough in China have local characteristics. Whether genes, geography and lifestyle diversity contribute to these differences is unknown. We have developed cough-related tests, such as the induced sputum test and capsaicin challenge test. With the release of the Chinese Guidelines on the Diagnosis and Treatment of Cough, we believe many more patients are being correctly diagnosed and receiving the appropriate treatment. However, the cause and pathogenesis of some types of chronic cough need to be further elucidated.

## Competing interests

The authors declare that they have no competing interests.

## Authors’ contributions

NSZ and KFL conceived the study. KFL and WL carried out the literature review and drafted the paper, GQZ drafted the paper and provided writing support. All authors read and approved the final manuscript.

## Authors’ information

KFL: M.D. PhD., Professor of Internal Medicine, State Key Laboratory of Respiratory Diseases, 1st Affiliated Hospital of Guangzhou Medical College, and PhD tutor of respiratory medicine.

WL: Master Degree in immunology, senior research fellow, State Key Laboratory of Respiratory Diseases, 1st Affiliated Hospital of Guangzhou Medical College.

GQZ: M.D., Associate Professor of Medicine, Professor of Editology and Publishing, State Key Laboratory of Respiratory Diseases, 1^st^ Affiliated Hospital of Guangzhou Medical College; Editorial Director, Journal of Thoracic Disease.

NSZ: Academician of Chinese Academy of Engineering, Professor of Internal Medicine, State Key Laboratory of Respiratory Diseases, 1st Affiliated Hospital of Guangzhou Medical College, and PhD tutor of respiratory medicine.
